# High Cognitive Ability and Mental Health: Findings from a Large Community Sample of Adolescents

**DOI:** 10.3390/jintelligence11020038

**Published:** 2023-02-18

**Authors:** Jeroen Lavrijsen, Karine Verschueren

**Affiliations:** Faculty of Psychology and Educational Sciences, KU Leuven, Tiensestraat 102 bus 3717, 3000 Leuven, Belgium

**Keywords:** giftedness, psychological adjustment, mental health

## Abstract

Whereas it has sometimes been asserted that the intellectually gifted would be more prone to develop mental health problems, empirical studies generally do not seem to support such claims. However, much of the available research has relied on preselected samples, introducing risks for sample selection bias. This study scrutinized the relationship between intellectual giftedness (defined as high cognitive ability) and mental health in a large, non-selective sample of early adolescents (*n* = 3409; 49.6% boys; M_age_ = 12.5 years). Using a standardized intelligence test (CoVaT-CHC) to identify participants with a high cognitive ability (IQ ≥ 120; *n* = 403), we compared self- and parent-reported levels of emotional problems, conduct problems, hyperactivity/inattention, and self-reported worry and global self-esteem between high and average ability adolescents. Findings indicated that adolescents with a high cognitive ability were not at increased risk of psychological maladjustment; if any, differences were in favor of the high ability group. However, adolescents who had been formally identified as gifted (i.e., who had received a gifted label) did report worse adjustment for a number of outcomes.

## 1. Introduction

The field of giftedness research has known a long-standing debate about the psychological adjustment of intellectually gifted youth. On one hand, gifted children and adolescents have sometimes been portrayed as being more vulnerable to mental health issues (e.g., Silverman 1994), as they struggle to find their place in a world that does not understand them. Such negative views about the psychological adjustment of intellectually gifted individuals are dominant among the general public; for example, a majority of German adults reported to see high cognitive ability as an obstacle to healthy emotional functioning (Baudson 2016), and Flemish trainee teachers anticipated hypothetical students to be less emotionally stable when they were described as intellectually gifted (Weyns et al. 2021). By contrast, empirical studies comparing the prevalence of psychological problems between gifted and non-gifted youth generally do not seem to confirm such negative stereotypes. Overall, these studies did not find indications that high cognitive ability would increase risks for psychological maladjustment; if any, differences were usually in favor of the intellectually gifted (Lein 2021; Neihart et al. 2002). However, in general, important methodological concerns have been raised about the quality of the research base (e.g., Zeidner 2021). In particular, sample selection practices, such as recruiting gifted participants from a preselected population (e.g., among students participating in summer schools or among gifted students seeking help from a counselor), have been argued to bias research findings either in a positive or negative way. To overcome these concerns and to advance our understanding of the psychological world of the intellectually gifted youth, this study investigated mental health in a large, non-selective sample of early adolescents, comparing an array of mental health indicators (i.e., global self-esteem, emotional problems, worry, conduct problems, and hyperactivity/inattention problems) between youth with high and with average cognitive ability. 

### 1.1. Intellectual Giftedness as High Cognitive Ability

In the literature, various definitions and models of giftedness have been proposed (e.g., Harder et al. 2014; Subotnik et al. 2011). Beyond their differences, contemporary models of giftedness share a number of common features. First, most models recognize that giftedness can manifest itself in many different domains (e.g., cognition, creativity, music). In this study, we focus on the cognitive or intellectual domain, which has been the most studied domain of giftedness (Jarosewich et al. 2002). Second, contemporary models of giftedness are developmental and contextual in nature, describing the process of talent development from high potential to outstanding achievement and identifying individual and environmental factors that influence this development (e.g., Gagné 2004; Preckel et al. 2020; Subotnik et al. 2011). Whereas several conceptions of intellectual giftedness have been proposed, sometimes broadening the concept of giftedness to include, for example, task commitment, creativity, practical intelligence, or ethical skills beyond high intellectual capacities (e.g., Sternberg 2017), this study restrictively defined intellectual giftedness as having high cognitive ability. Moreover, high cognitive ability has been measured in several ways, namely using teacher nominations, intelligence tests, achievement tests, portfolios, or combinations thereof. In this study and in line with the review by Francis et al. (2016) on giftedness and mental health, we used a standardized, curriculum-free cognitive test to assess adolescents’ cognitive ability (i.e., a standardized intelligence test). Standardized measures of intelligence are among the most widely used operationalizations of intellectual giftedness (Košir et al. 2016). Additionally, whereas academic achievement tests or teacher nominations may lead to an overrepresentation of high achieving participants and thus an underestimation of behavioral or emotional difficulties, such a selection bias is minimized when using curriculum-free cognitive tests (Francis et al. 2016). 

### 1.2. Intellectual Giftedness and Mental Health: Theoretical Expectations

Theoretically, a number of mechanisms have been proposed to explain that the intellectually gifted youth might be more vulnerable to psychological maladjustment. For example, some scholars have argued that intellectually gifted individuals possess certain traits, such as heightened emotional intensity or sensitivity, that would increase their vulnerability to psychological maladjustment (Lein 2021). Moreover, it has been argued that intellectually gifted children’s cognitive abilities develop at a faster rate than their emotional capabilities, a phenomenon called asynchronous development. When the socio-emotional development of intellectually gifted children does not keep pace with their cognitive functioning, this may lead to inner conflict, frustration, and anxiety. Others have argued that gifted children may feel different or alienated from their peers (Lein 2021), or that the environment of the intellectually gifted youth is sometimes not sufficiently responsive to their needs (e.g., Kanevsky and Keighley 2003). 

However, it has also been argued that advanced intellectual capacities may function as resources that make intellectually gifted youth more resilient to stressors in their environment (Neihart et al. 2002). For example, strong problem-solving skills may help intellectually gifted children and adolescents to better manage and solve internal and external conflicts. Similarly, intellectually gifted youth may better understand their emotions and thus be more effective in regulating them (Abdulla Alabbasi et al. 2020; Mayer et al. 2016). Hence, a high cognitive ability might also act as a resilience factor protecting young people from psychological maladjustment.

### 1.3. Comparing Mental Health between Intellectually Gifted and Non-Gifted Youth

Overall, empirical studies seem to be relatively positive about the psychological adjustment of intellectually gifted youth. Generally, differences in psychological adjustment between intellectually gifted and non-gifted individuals have been found to be negligible; if differences do emerge, these often favored intellectually gifted youth (Neihart 1999). Indeed, a meta-analysis summarizing nine studies concluded that anxiety disorders were less common in intellectually gifted youth, whereas rates of depression and suicidal ideation were comparable between gifted and non-gifted children and adolescents (Martin et al. 2010). Similarly, a systematic literature review of 18 studies found that intellectual giftedness was associated with lower levels of anxiety, behavioral problems, and general psychopathology (Francis et al. 2016). Finally, meta-analyses covering 40 studies published between 1997 and 2005 (Litster and Roberts 2011) and 36 studies published between 2005 and 2021 (Paniagua-Infantes et al. 2022) found that intellectually gifted children and adolescents reported, on average, somewhat higher levels of global self-esteem than their non-gifted peers. 

However, major concerns have been raised about the quality of the research on mental health in the intellectually gifted youth (Plucker and Callahan 2014; Zeidner 2021). Arguably, the main methodological concern still troubling gifted research today is the potential bias associated with the sample selection (Wiley 2020; Lein 2021). For reasons of convenience (i.e., recruiting a sufficiently large number of intellectually gifted individuals who are, by definition, relatively rare in the general population), studies have often recruited intellectually gifted participants from some kind of pre-selected subpopulation. For example, some studies have been based on reports by clinicians working with gifted youth. However, such studies may overestimate the incidence of emotional and behavioral problems in intellectually gifted youth, as students who see a clinician often do so because of a pre-existing psychosocial issue. Similarly, studies recruiting gifted participants among organizations for gifted people (e.g., Mensa) may be biased, as applications for memberships of such organizations may be associated with specific life experiences and needs of gifted individuals. In addition, the identification as being gifted could in itself influence subsequent psychosocial functioning, for example, because it may evoke fears of not living up to expectations or because it emphasizes being different from others (Freeman 2006). Other studies have recruited gifted children and adolescents among participants in specific educational programs for gifted students, such as pull-out programs and gifted summer schools. However, when particularly the most engaged and high-performing students are eager to or allowed to participate in such programs (Renzulli and Delcourt 1986), better-adjusted students might be overrepresented in such samples, implying that these studies would underestimate emotional or behavioral problems in the intellectually gifted youth. Still, other gifted programs may focus on remedying disengaged gifted students, which would lead to an overrepresentation of youth experiencing psychological maladjustment in the sample. In addition, gifted children and adolescents are often identified not through their scores on a valid intelligence test, but rather through teacher or parent nominations, which may also be biased, for example, towards better-adjusted children (Lavrijsen and Verschueren 2020). All in all, these different examples of sample selection practices emphasize that relying on preselected samples may bias findings either in a positive or in a negative direction, limiting the validity of the research base. Accordingly, due to the biases potentially associated with these practices, the reliance on convenience sampling has been identified as one of the main shortcomings of giftedness research (Francis et al. 2016; Subotnik et al. 2011). 

To date, only a few studies on the mental health of the intellectually gifted youth have investigated unselected samples, that is, adopted a design in which gifted participants were not preselected (e.g., by participation in a gifted program or gifted organization) but identified by administering a cognitive ability test to a large sample (Subotnik et al. 2011). For example, a German study (*n* = 760; mean age = 16.7 years) administered the Intelligence-Structure-Test 2000 R (Liepmann et al. 2007) to secondary school students to identify intellectually gifted students as those scoring in the top 2% of the test. This study found no differences between intellectually gifted and non-gifted adolescents in life satisfaction (Bergold et al. 2015), emotional stability (Wirthwein et al. 2019), and subjective well-being (Bergold et al. 2020). However, the sample in this study included only students attending the upper track of the German school system, implying that both intellectually gifted and non-gifted students were relatively high academic performers. Similarly, Dutch gifted primary school children (*n* = 513; mean age = 10.4 years; intellectually gifted students identified by scoring in the top 10% on the Dutch Intelligence Test for Educational Level, Van Dijk and Tellegen 2004) reported better socio-emotional functioning than their peers (Gubbels et al. 2018). However, this survey was confined to student functioning within the school context (e.g., well-being at school), thereby not covering the broader psychosocial functioning of gifted children. This indicates the need for large, non-selective studies to assess mental health in the intellectually gifted youth. 

### 1.4. The Present Study

This study compared mental health between intellectually gifted (defined as having high cognitive ability) and non-gifted early adolescents in a large, non-selective community sample of Flemish 7th graders (*n* = 3409; M_age_ = 12.5 years). The study considered several indicators of mental health, that is, global self-esteem, internalizing problems (emotional problems, worry), and externalizing problems (conduct problems, hyperactivity/inattention problems). All students were administered a cognitive ability test, allowing the comparison of mean levels of mental health indicators between participants with high and with average cognitive ability, controlling for social background and gender. As it has been argued that investigating mental health in adolescents benefits from a multi-informant approach (De Los Reyes et al. 2015), we surveyed, beyond student self-reports, also how parents evaluated mental health (i.e., emotional problems, conduct problems, and hyperactivity/inattention problems) in their child. 

Theoretically, explanations both for better and for worse psychological adjustment of youth with a high cognitive ability have been proposed. However, empirical studies have usually found equal or more favorable psychological adjustment in intellectually gifted adolescents compared to their non-gifted peers (Francis et al. 2016). Hence, we expected to find equal or higher global self-esteem and equal or lower levels of emotional problems, worry, conduct problems, and hyperactivity/inattention problems in students with a high cognitive ability.

In addition, we wanted to assess the degree to which a preselected subgroup of students, that is those previously identified as gifted (i.e., who had received a gifted label), would be representative for gifted adolescents in terms of mental health. Hence, we additionally compared mental health indicators between students formally identified as gifted and their non-identified peers. In this study, we expected worse psychological adjustment among students labeled as gifted, because in the Flemish context, this identification often occurs as part of a clinical assessment and interventions for at-risk children, implying the self-selection of students experiencing psychosocial difficulties into the labeled group.

## 2. Materials and Methods

### 2.1. Procedure and Participants

Data were collected within the TALENT-study (October–November 2017), a large-scale study among 3409 7th graders from 166 classes in 27 schools in Flanders, the Dutch-speaking part of Belgium (49.6% boys, M_age_ = 12.5 years). Selection bias was avoided by recruiting all students in the participating schools in the study, irrespective of prior achievement. Participants had a slightly more advantaged social background than the population of the general stream in Flemish secondary education, as 14.1% of them had a mother without a secondary school degree (18.0% in the population). Students were administered a cognitive ability test and completed a student survey in class. In addition, to obtain a multi-informant perspective on adolescent functioning, parents were surveyed. The study was approved by the Ethical Committee of KU Leuven. Prior to conducting the study, we obtained informed consent from adolescents and their parents. Missingness was low; 241 adolescents (7.6%) did not complete the cognitive ability test or student survey, yielding an analytic sample of 3168 students, whereas in addition for 352 adolescents (11.1%), none of the parents completed the parent survey, yielding an analytic sample for parent-reported mental health of 2816 adolescents. Little’s test indicated that missingness was not completely at random. In particular, parental response was lower for students from socially disadvantaged backgrounds; for example, the parental response rate among students receiving a school allowance was 78.5%, which was significantly lower (*p* < .001) than the rate among students not receiving such an allowance (87.8%). For other background characteristics (gender, cognitive ability), differences in parental response were smaller, although parents of female participants and parents of children with high cognitive ability were slightly more likely to return parental surveys. Student self-reported outcomes were less dependent on background characteristics; for example, survey response was roughly equal (*p* = .075) among students receiving an allowance (95.4%) and students not receiving an allowance (96.9%).

### 2.2. Measures

Adolescents completed a two-hour cognitive ability test (CoVaT-CHC; Magez et al. 2015) in class under the supervision of a trained member of the research team. The test built on the CHC-Model of intelligence (Horn and Cattell 1966) and assessed both fluid and crystallized intelligence. The test has demonstrated both content validity (Tierens 2015) and criterion validity (Magez and Bos 2015). An IQ-score (M = 100, SD = 15) for each student was calculated based on a comparison of test results with a representative norming sample. Adolescents were defined as having high cognitive ability if they scored among the top 10% of a representative age group (IQ between 120 and 130; *n* = 272) and as having very high cognitive ability if they scored among the top 2.5% of their age group (IQ above 130; *n* = 127). Students with high and with very high cognitive ability were compared to a reference group consisting of average ability students with an IQ between 90 and 110 (*n* = 1754) (this demarcation corresponds to the middle 50% of the intelligence distribution, excluding students with either particularly low or high cognitive ability). In addition, parents were asked to indicate whether their child had ever been formally identified as gifted. In Flanders, no large-scale system of ability testing is in place, but individual parents can have their child tested on demand. Children for whom parents indicated that they had been tested and had scored in the gifted range on an intelligence test (i.e., IQ above 120) were considered as being formally identified as gifted (*n* = 82).

Because participants in the larger project from which the current data were drawn filled out an extended battery of questionnaires, measures had to be as concise as possible. For Global Self-Esteem and Worry, measures were shortened by selecting the items that loaded highest on the respective constructs in factor analyses on previously collected independent samples. In particular, Students’ Global Self-Esteem was measured with 5 items from the Rosenberg Self-Esteem Scale (Rosenberg 1965). This is an established and widely validated scale (e.g., Franck et al. 2008) assessing students’ positive and negative evaluations of themselves in affective terms (e.g., *On the whole, I am satisfied with myself)*. Items were rated on an 5-point Likert scale; negative items were reverse coded. Internal consistency of the scale was high (α = .85). Worry was assessed with 5 items (e.g., *I am always worrying about something*) from the Penn State Worry Questionnaire for Children (PSWQ-C), measuring the tendency of children to engage in excessive, generalized, and uncontrollable worry. Validity of this scale has been established in adolescent samples (e.g., Muris et al. 2001), and internal reliability in the present study was excellent (α = .90). Emotional Problems (e.g., *I am often unhappy, down-hearted or tearful*), Conduct Problems (e.g., *I get very angry and often lose my temper*) and Hyperactivity/Inattention Problems (e.g., *I am restless, I cannot stay still for long*) were measured with the corresponding subscales (5 items each; not shortened) of the Strengths & Difficulties Questionnaire (Goodman 1997), a widely used scale to assess problem behavior in adolescents aged 11–17 years. In accordance with questionnaire guidelines, items were rated on a 3-point Likert scale. Parents were also administered the corresponding scales of the Strengths & Difficulties Questionnaire, assessing problematic behavior in their child (e.g., *My child is often unhappy, down-hearted or tearful*). Both parents were invited to complete the survey; if both parents responded, their ratings of child behavior were averaged. Correlations between mother and father ratings of students’ mental health were .65 (Emotional Problems), .54 (Conduct Problems), and .71 (Hyperactivity/Inattention Problems). For both student self-reports and parent-reports, internal consistencies of the Strengths & Difficulties Questionnaire scales were good for Emotional Problems (α_self_ = .74; α_parents_ = .75) and Hyperactivity/Inattention Problems (α_self_ = .68; α_parents_ = .78). For Conduct Problems, internal consistency was relatively low (α_self_ = .58; α_parents_ = .56). Relatively low reliability for the Conduct Problems subscale has been noted before (e.g., Di Riso et al. 2010; Muris et al. 2003), and probably reflects that the various problem behaviors assessed with the scale are somewhat heterogenous in nature (e.g., fighting, cheating, and stealing). To accommodate for lower internal consistency of the Conduct Problems scale, analyses for this scale were additionally performed at the item level. All analyses controlled for gender and social background, operationalized as maternal educational level (0: no secondary school degree, 1: at least secondary school degree).

### 2.3. Data Analysis

Before comparing adolescents with high and average cognitive ability, measurement invariance of the outcomes was investigated using Mplus. To ensure maximal use of the sample, a full information maximum likelihood (FIML) estimation was used. To establish measurement invariance, the model fit needs to be compared between three measurement models modeling each mental health outcome as a latent variable with the items as its manifest indicators within the average ability group (IQ 90–110) or within the high ability group (IQ above 120; for reasons of simplicity, students with high and with very high cognitive ability were grouped together for the analysis of measurement invariance). In Model A, loadings and intercepts were allowed to vary freely between both groups; Model B constrained loadings to be equal across groups, whereas intercepts were allowed to vary between groups; and Model C constrained both loading and intercepts to be equal across groups. For each mental health outcome and each measurement model, the comparative fit index (CFI), root mean square error of approximation (RMSEA), and standardized root mean square residual (SRMR) was calculated (we did not include χ^2^ as an index of model fit, as this index is known to be highly sensitive to sample size; Putnick and Bornstein 2016). Metric invariance of an outcome then can be established by comparing fit indices of Model B to Model A, whereas scalar invariance can be established by comparing the model fit of Model C to Model B. As a rule of thumb, invariance can be established when the decrease in CFI is not larger than 0.010 (Cheung and Rensvold 2002),when the increase in RMSEA is not larger than 0.010 (Chen 2007), or when the increase in SRMR is not larger than 0.025 (metric invariance) and 0.005 (scalar invariance), respectively (Chen 2007). 

## 3. Results

### 3.1. Preliminary Analyses

Table 1 reports the results of the preliminary analysis investigating measurement invariance of the various mental health indicators between the average and high ability group. Overall, measurement invariance was supported for most outcomes. Among the student self-reported mental health outcomes, both metric and scalar invariance were supported by at least two out of three fit indices for all outcomes, with the exception of conduct problems. For conduct problems, metric invariance was supported by all three fit indices, but scalar invariance was supported only by the small increase in the SRMR. This indication for limited measurement invariance of the conduct problems outcome adds to the limited internal consistency of this scale, which will be addressed by additional item-level analyses (see above). Among the parent-reported variables, both metric and scalar invariance was supported by at least two out of three fit indices for all outcomes, with the exception of hyperactivity/inattention problems. In addition, the level of model fit for this outcome was relatively low in all three measurement models. Accordingly, results for parent-reported hyperactivity/inattention problems should be treated with caution; anyhow, we will rely primarily on student self-reports to compare mental health between the average ability and high ability groups.^1^

### 3.2. Main Analyses

After establishing measurement invariance of most outcomes between ability groups, levels of mental health indicators were compared between adolescents with average, with high, and with very high cognitive ability, controlling for social background and gender. Table 2 reports mean level differences and Cohen’s *d* between these groups, whereas Table 3 reports latent means of the mental health indicators in the high and very high cognitive ability groups (latent means were fixed to zero among average ability respondents; hence, positive latent means indicate higher values in the high ability groups relative to the average ability group). Whereas latent means correct for measurement error, manifest scale means may be easier to interpret and compare with other studies. However, all results (i.e., whether statistically significant differences emerged between the high and average ability groups and whether these differences were positive or negative) were fully equivalent between both approaches (manifest/latent means). 

First, both adolescents with high and with very high cognitive ability reported significantly higher levels of global self-esteem. Regarding internalizing problems (emotional problems and worry), levels were not statistically distinguishable between the average ability group on one hand, and the high and very high cognitive ability group, on the other. Adolescents with a high cognitive ability reported less conduct problems and less hyperactivity and inattention problems than their average ability peers, whereas adolescents with a very high cognitive ability reported less conduct problems than their average ability peers. Given the low internal consistency of the measure of conduct problems, analyses for this scale were also performed at the item level. For all individual items, ratings were equal or lower in the high and very high cognitive ability groups relative to the average ability group^2^. Moreover, parents of adolescents with high and with very high cognitive ability confirmed that their children demonstrated less conduct problems and less hyperactivity and inattention, and a similar level of emotional problems, than average ability children (given the limited support for measurement invariance in the parent-reported measures, these results should be treated with some caution). Effect sizes of significant differences sizes were typically close to *d* = 0.20 for the manifest scales and *d* = 0.30 for the latent means, which both correspond to relatively weak effects (the larger effect sizes in the case of parent-reported hyperactivity and inattention problems should be treated with caution due to limited measurement invariance of this variable, see above).

Finally, Table 4 compares mental health outcomes between adolescents with and without a formal identification as gifted. Adolescents labeled as gifted reported lower global self-esteem and higher levels of emotional problems, worry, and hyperactivity/inattention than their non-labeled peers, whereas levels of conduct problems were not statistically distinguishable between both groups. Similarly, parents of adolescents labeled as gifted reported higher levels of emotional problems and conduct problems than parents of non-labeled adolescents; levels of hyperactivity/inattention were not statistically distinguishable between both groups.

## 4. Discussion

Whereas intellectually gifted adolescents have sometimes been supposed to be at an increased risk for mental health problems, this study refutes such negative claims. In a non-selective sample, adolescents with a high cognitive ability were, on average, more satisfied with themselves (higher global self-esteem) than their average ability peers, reported lower levels of externalizing problems (conduct problems, hyperactivity/inattention) than their average ability peers, and were found to be similar to their average ability peers in terms of internalizing problems (emotional problems, worry). Moreover, adopting a multi-informant approach to complement student self-report with a parental perspective (De Los Reyes et al. 2015), parents confirmed this overall positive account of the psychological adjustment of adolescents with a high cognitive ability. Overall, findings were similar when considering adolescents with a high cognitive ability (i.e., IQ between 120 and 130) and adolescents with a very high cognitive ability (i.e., IQ above 130). These findings add to an increasing body of knowledge (Martin et al. 2010; Francis et al. 2016; Paniagua-Infantes et al. 2022) that high cognitive ability is not in itself a risk factor for psychological maladjustment, and that in some cases (e.g., externalizing problems), it might even be a resilience factor shielding youth from mental health issues. Whereas this study did not investigate which mechanisms drive this remarkably possible account of the psychological world of adolescents with a high cognitive ability, it could be that their advanced problem-solving skills help these adolescents to understand and efficiently manage potential conflicts, thus taking the sting out of issues that might otherwise escalate into major behavioral problems (Abdulla Alabbasi et al. 2020). Further research is needed to investigate why high cognitive ability is often related to better psychological adjustment, and to inform further theory development on this issue. 

The finding that adolescents with a high cognitive ability are not particularly vulnerable to psychological maladjustment may be reassuring for young people and their parents. Moreover, that mental health issues are not typical for high ability students strongly suggests that their identification should not depend on the presence of particular emotional or psychological problems. Indeed, ill-informed assumptions associating high cognitive ability with psychological problems may disrupt the proper identification of intellectually gifted youth. For example, when teachers expect high ability students to struggle with psychological problems (e.g., worry), they may overlook the cognitive talent of students with a high cognitive ability who do not exhibit such problems.

Of course, even when, on average, high cognitive ability is unrelated to psychological problems, some high ability adolescents may still encounter mental health problems. Further research is needed to pin down the factors that make these high ability youngsters vulnerable to psychological maladjustment. For example, general developmental research has identified a number of contextual risk factors for maladjustment in the full population, related to the quality of relationships with parents, teachers, and peers (e.g., McLeod et al. 2007a, 2007b). To date, only a few studies have investigated whether these risk factors also apply to the subgroup of adolescents with a high cognitive ability. For example, authoritarian and controlling parenting has been associated with maladaptive outcomes such as anxiety and perfectionism, also among gifted youth (Dwairy 2004; Lavrijsen et al. 2021; Tam and Phillipson 2013). Similarly, a poor fit between the needs of high ability students and their school environment (e.g., underchallenging instruction) could contribute to reduced school motivation and school well-being, possibly increasing risks of psychological maladjustment (Mueller and Winsor 2018; Ramos et al. 2021). However, more research is needed to further specify how the environment of adolescents with a high cognitive ability could help them to be more resilient towards mental health threats. 

A particular contribution of this study is that it emphasized the importance of non-selective samples to investigate mental health in intellectually gifted adolescents (Wiley 2020; Lein 2021). Indeed, the reliance on pre-selected samples (i.e., recruiting participants from subpopulations already identified as gifted) has been recognized as one of the major shortcomings of the research on intellectual giftedness (Subotnik et al. 2011). The present study showed that whereas adolescents scoring high on a cognitive ability test reported similar or lower levels of mental health issues than their average ability peers, adolescents formally identified as gifted (i.e., those who had received a gifted label independent of the study) did report an elevated risk for emotional problems, worry, hyperactivity, and inattention problems, and were generally less satisfied with themselves (lower self-esteem) than their non-identified peers. This discrepancy between measured high cognitive ability and identified giftedness echoes findings by Freeman (2006), who reported that identified gifted children had more emotional problems than a control sample of equally able, but not formally identified children. Our cross-sectional study does not allow the establishment of the direction of this association between identification as gifted and reduced mental health. In one way, this could be a selection effect. In Flanders, there is no systematic ability testing in place; rather, the identification as gifted typically occurs as part of individual counselling trajectories for children experiencing academic or psychosocial difficulties. Indeed, cognitive tests are often administered as part of the assessment of the child’s problem: when these tests then indicate high cognitive ability, the child is formally identified as gifted. However, when children are often identified as gifted because of participating in counselling trajectories, children with pre-existing psychological difficulties may be overrepresented in the labeled group. The other way round, being formally identified as gifted could also impact the psychosocial functioning of labeled adolescents; for example, it might be a clear sign (for themselves and for others) that there is something inherently “special” about them, increasing feelings of being different and isolated from peers (Freeman 2006). Moreover, the gifted label may evoke fears in adolescents that they will not be able to meet the exaggerated expectations that they or their environment may associate with having outstanding cognitive abilities. Whereas the present study cannot distinguish between both directions, the finding, that adolescents identified as gifted are not representative of the population of adolescents with a high cognitive ability, highlights that findings from studies recruiting intellectually gifted youth among some formally identified subpopulation (e.g., gifted adolescents seeing a clinician, students participating in a gifted program, members of gifted organizations) might not be generalizable to the mental health of gifted youth as a whole. Accordingly, future research should remain cautious to avoid sample selection bias.

Beyond the many strengths of this study, such as the large, non-selective sample, the use of a validated cognitive ability test to identify high ability adolescents, and the adoption of a multi-informant perspective complementing student self-ratings with parental evaluations, some limitations of the study have to be acknowledged. First, the study was cross-sectional in nature, which impeded investigating the development of mental health over time. A particular issue for further research would be to disentangle selection effects from labeling effects to explain the worse psychological adjustment among students identified as gifted—an association that is contrasted with the equal or more favorable adjustment among children with measured high cognitive ability. A longitudinal study, following children over time, would be better suited to pin down the psychological consequences of receiving a gifted label. Second, whereas we investigated mental health in adolescents with a very high cognitive ability (i.e., those scoring in the top 2.5% of their age group), we did not further differentiate to see whether those with exceptionally high cognitive abilities (e.g., IQ above 150) would be more prone to mental health issues. It has sometimes been suggested that for individuals with exceptionally high cognitive ability, the extremely large cognitive distance to their peers could cause feelings of alienation, isolation, and related psychosocial problems (Shaywitz et al. 2001). However, in a non-selective sample, participants with a exceptionally high cognitive ability are, by definition, very rare (e.g., in the population, only 1 out of 2000 children score an IQ above 150); samples would thus have to be very large to investigate whether exceptional cognitive abilities would be related to higher risks for psychological maladjustment. Third, this study restrictively defined “intellectual giftedness” as having high cognitive ability. Some contemporary theorists of giftedness have advocated a broader conception of giftedness, seeing high cognitive ability as only one of many components of giftedness. For example, according to Sternberg’s (2017) giftedness model, individuals must also display high creative, practical, wisdom-based, and ethical skills in order to qualify as gifted. When giftedness would be conceptualized as such a larger set of capacities, beyond high cognitive ability, findings on the mental health of gifted youth may be different from the ones reported in the present study. Furthermore, the present study identified high ability adolescents as those having a high general cognitive ability. Recent models of talent development (e.g., Preckel et al. 2020) advocate a more differentiated view on abilities, emphasizing the importance of domain-specific abilities (e.g., mathematical talent), each with their own developmental trajectories (e.g., relatively early peaks for mathematical talent). Future research could consider whether there are particular vulnerabilities associated with scoring high on a certain domain-specific ability, beyond general intelligence. Finally, whereas the study controlled for social background and gender, missingness (in particular of parental reports of student mental health) was somewhat higher among students from low social backgrounds. Further research should further test the generalizability of our results.

## 5. Conclusions

In a large non-selective sample of 3409 seventh grade students, five indicators of mental health were compared between adolescents with a high cognitive ability and their average ability aged peers. Adolescents scoring among the top 10% or top 2.5% of their age group on a cognitive ability test were not found to report more mental health problems than their peers; if any, differences were in favor of the high ability groups. However, students who had been formally identified as gifted (i.e., who had received a gifted label) did report worse adjustment for a number of mental health indicators. 

## Figures and Tables

**Table 1:** Measurement invariance of mental health indicators between high ability (IQ ≥ 120) and average ability (90 ≤ IQ ≤ 110) adolescents. Difference values in bold indicate that the observed changes in model fit supported measurement invariance (decrease in CFI ≤ 0.010; increase in RMSEA ≤ 0.010; increase in SRMR ≤ 0.025 (metric invariance) or ≤0.005 (scalar invariance)).

Model A: Free Loadings and Intercepts		Model B: Loadings Constrained to Be Equal across Groups		Model C: Loadings and Intercepts Constrained to Be Equal across Groups	
	Value	Value	Diff. B-A	Value	Diff. C-B
**Student self-report**					
Global Self-Esteem					
CFI	0.951	0.952	**0.001**	0.944	**−0.008**
RMSEA	0.146	0.117	**−0.029**	0.108	**−0.009**
SRMR	0.034	0.038	**0.004**	0.075	0.037
Emotional Problems					
CFI	0.979	0.978	**−0.001**	0.969	**−0.009**
RMSEA	0.066	0.054	**−0.012**	0.055	**0.001**
SRMR	0.024	0.030	**0.006**	0.033	**0.003**
Worry					
CFI	0.951	0.952	**0.001**	0.951	**−0.001**
RMSEA	0.127	0.147	0.020	0.127	**−0.020**
SRMR	0.038	0.034	**−0.004**	0.038	**0.004**
Conduct Problems					
CFI	0.951	0.943	**−0.008**	0.912	−0.031
RMSEA	0.075	0.063	**−0.012**	0.065	**0.002**
SRMR	0.028	0.036	**0.008**	0.046	0.010
Hyperactivity/Inattention					
CFI	0.879	0.879	**−0.000**	0.863	−0.016
RMSEA	0.136	0.120	**−0.016**	0.105	**−0.015**
SRMR	0.049	0.051	**0.002**	0.056	**0.005**
**Parent report**					
Emotional Problems					
CFI	0.984	0.981	**−0.003**	0.968	−0.013
RMSEA	0.061	0.056	**−0.005**	0.063	**0.007**
SRMR	0.019	0.027	**0.008**	0.030	**0.003**
Conduct Problems					
CFI	0.958	0.905	−0.053	0.883	−0.022
RMSEA	0.062	0.070	**0.008**	0.066	**−0.004**
SRMR	0.025	0.050	**0.025**	0.055	**0.005**
Hyperactivity/Inattention					
CFI	0.844	0.833	−0.011	0.808	−0.025
RMSEA	0.235	0.203	**−0.032**	0.187	**−0.016**
SRMR	0.069	0.074	**0.005**	0.092	0.018

**Table 2:** Comparison of mental health indicators in adolescents with high and very high cognitive ability vs. adolescents with average cognitive ability.

Measure	Average Ability (90 ≤ IQ ≤ 110)		High Cognitive Ability (120 ≤ IQ < 130)				Very High Cognitive Ability (130 ≤ IQ)			
	Mean	SE	Mean	SE	*p*	*d*	Mean	SE	*p*	*d*
**Student self-report**										
Global Self-Esteem	3.76	0.85	**3.95**	**0.78**	**<.001**	**0.22**	**3.97**	**0.72**	**.007**	**0.25**
Emotional Problems	1.55	0.47	1.53	0.48	.570	−0.04	1.58	0.47	.406	0.06
Worry	2.39	1.04	2.33	0.98	.454	−0.06	2.38	1.10	.963	−0.01
Conduct Problems	1.33	0.31	**1.27**	**0.27**	**<.001**	**−0.19**	**1.25**	**0.25**	**.004**	**−0.26**
Hyperactivity/Inattention	1.81	0.45	**1.73**	**0.44**	**.014**	**−0.18**	1.80	0.46	.877	−0.02
**Parent report**										
Emotional Problems	1.39	0.38	1.37	0.36	.458	−0.05	1.34	0.34	.108	−0.13
Conduct Problems	1.18	0.22	**1.14**	**0.18**	**.003**	**−0.18**	**1.14**	**0.17**	**.047**	**−0.18**
Hyperactivity/Inattention	1.59	0.46	**1.43**	**0.38**	**<.001**	**−0.35**	**1.35**	**0.40**	**<.001**	**−0.52**

Note. Significant differences with average ability group in bold. Range [1–5] for Global Self-Esteem and Worry, [1–3] for Emotional Problems, Conduct Problems, and Hyperactivity/Inattention.

**Table 3:** Latent means of mental health indicators in adolescents with high and with very high cognitive ability. Latent means fixed to zero for average ability adolescents.

Measure	High Cognitive Ability (120 ≤ IQ < 130)				Very High Cognitive Ability (130 ≤ IQ)			
	Mean	SE	*p*	*d*	Mean	SE	*p*	*d*
**Student self-report**								
Global Self-Esteem	**0.22**	**0.05**	**<.001**	**0.29**	**0.24**	**0.06**	**<.001**	**0.31**
Emotional Problems	−0.03	0.03	.339	−0.09	0.01	0.04	.727	0.03
Worry	−0.07	0.06	.268	−0.07	−0.06	0.09	.522	−0.06
Conduct Problems	**−0.04**	**0.01**	**<.001**	**−0.26**	**−0.04**	**0.00**	**.002**	**−0.26**
Hyperactivity/Inattention	**−0.10**	**0.03**	**.002**	**−0.27**	−0.02	0.04	.712	−0.05
**Parent report**								
Emotional Problems	−0.01	0.02	.575	−0.04	−0.04	0.02	.077	−0.17
Conduct Problems	**−0.06**	**0.02**	**<.001**	**−0.20**	**−0.05**	**0.02**	**.006**	**−0.16**
Hyperactivity/Inattention	**−0.16**	**0.03**	**<.001**	**−0.41**	**−0.19**	**0.04**	**<.001**	**−0.49**

Note. Significant differences with average ability group in bold.

**Table 4:** Comparison of mental health indicators between non-labeled adolescents and adolescents formally labeled as gifted.

Measure	Not Formally Labeled as Gifted		Formally Labeled as Gifted			
	Mean	SE	Mean	SE	*p*	*d*
**Student self-report**						
Global Self-Esteem	3.79	0.84	**3.57**	**1.04**	**.022**	**−0.26**
Emotional Problems	1.55	0.48	**1.73**	**0.56**	**<.001**	**0.38**
Worry	2.38	1.04	**2.78**	**1.25**	**<.001**	**0.38**
Conduct Problems	1.33	0.30	1.39	0.32	.091	0.20
Hyperactivity/Inattention	1.80	0.45	**1.91**	**0.55**	**.026**	**0.24**
**Parent report**						
Emotional Problems	1.41	0.39	**1.59**	**0.47**	**<.001**	**0.46**
Conduct Problems	1.20	0.22	**1.28**	**0.25**	**.002**	**0.36**
Hyperactivity/Inattention	1.60	0.45	1.61	0.54	.736	0.02

Note. Significant differences with average ability group in bold. Range [1–5] for Global Self-Esteem and Worry, [1–3] for Emotional Problems, Conduct Problems, and Hyperactivity/Inattention.

## Data Availability

The data presented in this study are available on request from the corresponding author. The data are not publicly available due to privacy restrictions.

## Notes

1 As discussed by Yoon and Lai (2018), imbalances in subgroup size might affect the results of measurement invariance testing. Hence, we additionally tested measurement invariance contrasting the high ability group with a reference group restricted to students with an IQ between 98 and 102, which consists of 469 students and thus, is similar in size to the high ability group (*n* = 444). Results are reported in the Supplementary Materials (Table S1). By and large, results for self-reported mental health outcomes were similar to the findings reported for the analysis with the full reference group. In particular, metric invariance was supported (by at least two out of three fit indices) for all self-reported outcomes, with the exception of conduct problems; scalar invariance was supported for all self-reported outcomes except conduct and hyperactivity problems. For the parent-reported outcomes, scalar invariance could not be established, suggesting to treat results for these variables with caution, primarily building on student self-report to compare mental health between the average and high ability groups.

2 In particular, for the item ”*I usually do as I am told*”, [inverted] levels were not statistically distinguishable between the average, high, and very high ability groups; for the items “*I take things that are not mine from home, school or elsewhere*” and “*I fight a lot. I can make other people do what I want*”, levels were significantly lower in high and very high ability groups relative to the average ability group; for the item “*I get very angry and often lose my temper”*, levels were lower in the high ability group and not statistically distinguishable from the average ability group in the very high ability group; for the item “*I am often accused of lying or cheating”*, levels were lower in the very high ability group and not statistically distinguishable from the average ability group in the high ability group.

## Supplementary Materials

The following supporting information can be downloaded at: https://www.mdpi.com/article/10.3390/jintelligence11020038/s1, Table S1. Measurement invariance of mental health indicators between high ability and average ability adolescents, with the average ability group restricted to IQ between 98 and 102, balancing sample sizes between groups. Difference values in bold indicate that the observed changes in model fit supported measurement invariance (decrease in CFI ≤ 0.010; increase in RMSEA ≤ 0.010; increase in SRMR ≤ 0.025 (metric invariance) or ≤0.005 (scalar invariance)).
