# Supplementary material for: High Cognitive Ability and Mental Health: Findings from a Large Community Sample of Adolescents
Source: J Intell. 2023 Feb 18;11(2):38. doi: 10.3390/jintelligence11020038 (PMC9966861; doi:10.3390/jintelligence11020038)
Supplement: Supplementary file 1 [file jintelligence-11-00038-s001.zip › jintelligence-2100149-supplementary.pdf]

Table S1. Measurement invariance of mental health indicators between high ability and average ability adolescents, with the average ability group restricted to IQ between 98 and 102, balancing sample sizes between groups. Difference values in bold indicate that the observed changes in model fit supported measurement invariance (decrease in CFI ≤ 0.010; increase in RMSEA ≤ 0.010; increase in SRMR ≤ 0.025 (metric invariance) or ≤ 0.005 (scalar invariance)).

	Model A: Free loadings and intercepts	Model B: Loadings constrained to be equal across groups		Model C: Loadings and intercepts constrained to be equal across groups	
	Value	Value	Diff. B-A	Value	Diff. C-B
Student self-report					
Global Self-Esteem					
CFI	0.930	**0.931**	**0.001**	**0.925**	**-0.006**
RMSEA	0.170	**0.138**	**-0.032**	**0.127**	**-0.011**
SRMR	0.058	**0.068**	**0.010**	0.083	0.015
Emotional Problems					
CFI	0.965	**0.969**	**0.004**	0.957	-0.012
RMSEA	0.082	**0.063**	**-0.019**	**0.066**	**0.003**
SRMR	0.034	**0.037**	**0.003**	**0.042**	**0.005**
Worry					
CFI	0.948	**0.948**	**-0.000**	**0.947**	**-0.001**
RMSEA	0.171	**0.142**	**-0.029**	**0.131**	**-0.011**
SRMR	0.040	**0.047**	**0.007**	**0.050**	**0.003**
Conduct Problems					
CFI	0.991	0.979	-0.012	0.910	-0.069
RMSEA	0.028	**0.034**	**0.006**	0.060	0.026
SRMR	0.023	**0.034**	**0.011**	0.062	0.028
Hyperactivity/Inattention					
CFI	0.892	**0.895**	**0.003**	0.862	-0.033
RMSEA	0.128	**0.105**	**-0.023**	**0.110**	**0.005**
SRMR	0.053	**0.055**	**0.002**	0.070	0.015
Parent report					
Emotional Problems					
CFI	0.979	**0.982**	**0.003**	0.956	-0.026
RMSEA	0.076	**0.056**	**-0.02**	0.074	0.018
SRMR	0.026	**0.032**	**0.006**	0.042	0.010
Conduct Problems					
CFI	0.964	0.929	-0.035	0.885	-0.044
RMSEA	0.047	**0.050**	**0.003**	**0.055**	**0.005**
SRMR	0.028	**0.043**	**0.015**	0.059	0.016
Hyperactivity/Inattention					
CFI	0.775	**0.778**	**0.003**	0.734	-0.044
RMSEA	0.288	**0.229**	**-0.059**	**0.215**	**-0.014**
SRMR	0.086	**0.087**	**0.001**	0.131	0.044
